# 3D-cultured hADSCs-derived exosomes deliver circ_0011129 to synergistically attenuate skin photoaging

**DOI:** 10.3389/fgene.2025.1627472

**Published:** 2025-09-03

**Authors:** Yu Zhang, Feng Zhou, Gang Nie, Liang Li, Juan Wen, Shimin He, Amin Yao

**Affiliations:** ^1^ Department of Dermato-Venereology, The Seventh Affiliated Hospital of Sun Yat-sen University, Shenzhen, China; ^2^ Department of Dermato-Venereology, The Third Affiliated Hospital of Sun Yat-sen University, Guangzhou, China; ^3^ Pediatric Hematology Laboratory, Division of Hematology/Oncology, Department of Pediatrics, The Seventh Affiliated Hospital of Sun Yat-sen University, Shenzhen, China; ^4^ Department of Dermato-Venereology, Dermatology Hospital, Southern Medical University, Guangzhou, China

**Keywords:** skin photoaging, exosomes, circ_0011129, hADSCs, collagen, elastin

## Abstract

**Background:**

Skin photoaging is primarily induced by ultraviolet (UV) exposure, involving mechanisms such as reactive oxygen species (ROS) accumulation, matrix metalloproteinase (MMP)-mediated collagen degradation, and cathepsin (e.g., Cathepsin K)-driven elastin denaturation and aggregation. Although circular RNA (circRNA) shows significant potential in regulating skin photoaging, its clinical translation remains challenging due to poor *in vivo* stability and targeted delivery efficiency.

**Objective:**

This study aimed to construct a 3D-cultured human adipose-derived mesenchymal stem cell (hADSC)-derived exosome (3D-Exo) loaded with circ_0011129 (3D-circ-Exo) and investigate its protective effects and molecular mechanisms against chronic UV-induced damage in human dermal fibroblasts (HDFs).

**Methods:**

A circ_0011129-overexpressing hADSC cell line was established via lentiviral transfection. Exosomes were isolated, and circRNA integrity was validated through divergent/convergent primer amplification, sequencing, and RNase R digestion. A chronic photoaging HDFs model was induced by 7-day UVA irradiation (5 J/cm^2^/d). Cellular senescence (SA-β-gal staining, p53/p21/p16 expression) and extracellular matrix degradation (collagen I, elastin) were assessed. Therapeutic effects were evaluated across four groups: light-shielded control, UVA-irradiated control, 3D-Exo + UV, and 3D-circ-Exo + UV.

**Results:**

The 3D-circ-Exo carrier successfully encapsulated circ_0011129 with a closed circular structure and significantly higher stability than linear RNA (p < 0.001). In the chronic photoaging model, UVA irradiation increased SA-β-gal-positive cells (p < 0.01), upregulated p53/p21/p16 protein expression (p < 0.01), and reduced collagen I and elastin levels (p < 0.001). Compared to 3D-Exo, 3D-circ-Exo demonstrated superior anti-photoaging effects: reduced SA-β-gal-positive cells (p < 0.05), downregulated p53/p21/p16 (p < 0.01), and restored collagen I/elastin expression (p < 0.01), significantly outperforming 3D-Exo.

**Conclusion:**

By integrating 3D culture with exosome delivery technology, this study constructed a functionalized circ_0011129 carrier (3D-circ-Exo) for the first time. 3D-circ-Exo significantly enhances anti-photoaging efficacy compared to 3D-Exo, suggesting that 3D-cultured exosomes synergize with circ_0011129 to inhibit cell cycle arrest (p53/p21/p16) and counteract UV-induced collagen loss and elastin denaturation. This work provides an innovative strategy for clinical photoaging intervention.

## 1 Introduction

Up to 80% of accelerated skin aging is attributed to chronic and excessive ultraviolet (UV) exposure, a phenomenon termed skin photoaging (Pannakal et al., 2025; Hajialiasgary Najafabadi et al., 2024). Skin photoaging refers to the premature aging of skin induced by chronic ultraviolet (UV) exposure, characterized by dysregulation of extracellular matrix metabolism (e.g., collagen degradation, elastin abnormalities), cellular senescence, and accumulated DNA damage. As the primary cause of extrinsic skin aging, photoaging manifests as skin laxity, wrinkles, and pigmentation. Furthermore, it is closely linked to sunlight-related dermatoses (e.g., actinic keratosis) and cutaneous malignancies (e.g., melanoma) (Kimura et al., 2025; Wenande et al., 2025; Zundell et al., 2025). Consequently, elucidating photoaging mechanisms and developing effective interventions are clinically important.

Our prior research identified Cathepsin K as a key effector in UV-induced elastin degradation, regulated by the MAPK/AP-1 signaling pathway (Xu et al., 2014), suggesting that simultaneous modulation of collagen and elastin metabolism may represent an effective therapeutic strategy. UV radiation induces reactive oxygen species (ROS) accumulation, pro-inflammatory cytokine release, and DNA damage in dermal fibroblasts (HDFs), disrupting their function (Budluang et al., 2025; Sarandy et al., 2024). UV also upregulates matrix metalloproteinases (MMPs, e.g., MMP1, MMP3, MMP9), which degrade collagen, and activates cathepsin K, driving elastin denaturation and abnormal aggregation (Gopalakrishnan et al., 2025; Zheng et al., 2017). While MMPs are central mediators of photoaging, they do not fully explain pathological changes in elastic fibers. We previously identified cathepsin K as a key effector in UV-induced elastin degradation, regulated by the MAPK/AP-1 signaling pathway (Xu et al., 2014), suggesting that simultaneously targeting collagen and elastin metabolism represents a promising therapeutic strategy.

Non-coding RNAs, particularly circular RNAs (circRNAs), have emerged as pivotal disease regulators. Formed by back-splicing into covalently closed loops, circRNAs exhibit nuclease resistance and greater stability than other non-coding RNAs, enhancing their therapeutic potential (Vi et al., 2023; Koyasu et al., 2023). We first discovered that circ_0011129 (circCOL-ELNs) regulates collagen synthesis and elastin stability through sponging miR-6732-5p, mitigating UVA-induced collagen loss and elastin degeneration in HDFs (Peng et al., 2018; Lin et al., 2020; Zhang et al., 2022). However, circRNA instability and inefficient *in vivo* targeted delivery severely limit its clinical application.

Exosomes, natural nanoscale vesicles, are ideal RNA delivery vehicles due to their low immunogenicity, stability, and intrinsic targeting capacity (Liang et al., 2022; Bernardi and Balbi, 2020). Notably, exosomes derived from adipose-derived mesenchymal stem cells (hADSCs) can act as drug carriers and secrete anti-inflammatory and growth factors that counteract UV-induced damage (Wang et al., 2024; Liu et al., 2024). Critically, hADSCs were prioritized over alternative sources (e.g., bone marrow, placental MSCs) due to superior clinical accessibility: subcutaneous adipose tissue from liposuction provides abundant donor material with minimal invasiveness, low immunogenicity, and no ethical concerns, contrasting with bone marrow aspiration limitations and placental heterogeneity issues. Recent advances in three-dimensional (3D) culture significantly enhance exosome yield and functionality (Pamulang et al., 2025; Zhong et al., 2025; Lee and Lee, 2024). For instance, exosomes from 3D-cultured mesenchymal stem cells show superior therapeutic efficacy in models of Alzheimer’s disease and skin photoaging (Shah et al., 2024; Kee et al., 2025; Cui et al., 2025). Building on these findings, we hypothesize that integrating 3D culture into exosome delivery systems to generate functionalized exosomes loaded with circ_0011129 (3D-circ-Exo) may overcome current photoaging intervention limitations through synergistic regulation of collagen metabolism and elastin homeostasis. This study aims to determine whether 3D-circ-Exo overcomes RNA delivery limitations enabling stable circ_0011129 delivery and enhanced photoprotection, and to investigate if it provides synergistic effects by simultaneously modulating cell cycle arrest (via p53/p21/p16) and extracellular matrix metabolism (collagen/elastin homeostasis). Addressing these questions will validate the therapeutic potential of engineered exosomes for RNA delivery and pioneer an integrated approach targeting the complex pathogenesis of cutaneous photoaging, potentially overcoming current single-target limitations with a novel multi-target strategy for clinical management.

## 2 Materials and methods

### 2.1 Isolation, identification, and differentiation of hADSCs

Subcutaneous adipose tissue was obtained from female liposuction patients (aged 25–45 years) with informed consent and approval from the Ethics Committee of the Seventh Affiliated Hospital of Sun Yat-sen University (No. KY-2022-024-03). Primary hADSCs were isolated from each donor. Tissue was digested with 1% collagenase I (Sigma-Aldrich, St. Louis, MO, United States) at 37°C for 40 min. After removing connective tissue, cell pellets were resuspended in DMEM-F/12 medium (BI Israel Beit Haemek Ltd., Kibbutz Beit Haemek, Israel) containing 20% fetal bovine serum (FBS, BI Israel Beit Haemek Ltd., Kibbutz Beit Haemek, Israel) and cultured in T25 flasks (Corning Incorporated, Corning, NY, United States) at 37 °C with 5% CO_2_. Adipogenic and osteogenic differentiation potential was assessed using induction kits (CytoNiche Biotech, Tianjin, China).

### 2.2 Cell lines and culture

Human dermal fibroblasts (HDFs) were isolated from foreskin tissue of healthy boys (5–10 years old) undergoing circumcision at the Third Affiliated Hospital of Sun Yat-sen University (with guardian consent). Primary HDFs were isolated from each donor. Tissue was digested with 0.25% trypsin (Gibco, Thermo Fisher Scientific, Waltham, MA, United States) and cultured in DMEM medium (BI Israel Beit Haemek Ltd., Kibbutz Beit Haemek, Israel) containing 10% FBS (ExCell Bio, Shanghai, China) and 1% penicillin/streptomycin (Corning Incorporated, Corning, NY, United States). hADSCs were cultured in DMEM-F/12 medium with 10% FBS, 2% penicillin/streptomycin, and 10 ng/mL basic fibroblast growth factor. hADSCs were cultured in three dimensions (3D) using microcarrier-based FloTrix miniSpin bioreactors (CytoNiche Biotech, Tianjin, China) according to the manufacturer’s protocol.

### 2.3 Exosome isolation

Exosomes (sEVs) were isolated via differential centrifugation: cell culture supernatant was sequentially centrifuged at 500×g (10 min, 4°C), 2,000×g (20 min, 4°C), and 10,000×g (40 min, 4°C) to remove cells and debris. The supernatant was ultracentrifuged at 120,000×g (70 min, 4°C) using an Optima XE or similar ultracentrifuge (Beckman Coulter, Brea, CA, United States), and the exosome pellet was resuspended in PBS and stored at −80°C.

### 2.4 Plasmid construction and stable cell line establishment

The circ_0011129 overexpression plasmid (pLC5-ciR-puro-0011129-GFP) was purchased from Guangzhou Jisai Biotechnology Co., Ltd. (Guangzhou, China). Plasmid functionality was validated by transfecting HEK-293T cells (70%–80% confluence) using Lipofectamine™ 3000 (Thermo Fisher Scientific, Waltham, MA, United States); circ_0011129 overexpression was confirmed by qPCR using a LightCycler^®^ 96 System (Roche Diagnostics, Basel, Switzerland) 48 h post-transfection. Lentivirus (Hanheng Biotechnology, Shanghai, China) was used to transfect 3D-hADSCs. Stable cells were selected with puromycin (2 μg/mL). circ_0011129 expression was validated by qPCR in blank control (NC), empty vector (pLC5-ciR), and overexpression vector (pLC5-circ_0011129) groups.

### 2.5 Validation of 3d-circ-exo

(1) Circular Structure Verification: RNA extracted from 3D-circ-Exo was reverse-transcribed to cDNA. Divergent and convergent primers were used to amplify circ_0011129, followed by agarose gel electrophoresis (2%) and Sanger sequencing (Aiji Biotech, Guangzhou, China) to validate circularization sites.
(2) Stability Verification: Total RNA was treated with RNase R (20 U/μg, Epicentre) for 30 min circ_0011129 and its linear isoform (Linear_0011129) were quantified via qPCR.
(3) Overexpression Verification: circ_0011129 levels in 3D-circ-Exo and empty vector 3D-Exo were compared using qPCR (primers in Table 1).

**TABLE 1:** The sequences of the qPCR primers.

Gene	Forward primer sequence 5′→3′	Reverse primer sequence 5′→3′
GAPDH-Circ	ATG​GCC​TCC​AAG​GAG​TAA​ATG	AGG​TCA​ATG​AAG​GGG​TCA​TTG
GAPDH-Linear	AGA​AGG​CTG​GGG​CTC​ATT​TG	GCA​GGA​GGC​ATT​GCT​GAT​GAT
circ_0011129	GCT​TTG​TGG​AAG​ACC​CTA​CT	CAC​TGC​CAG​GTT​GTC​TAC​T
Linear_0011129	TAC​ATT​GCC​ATC​ATG​GCT​GC	CTG​AGT​TTG​CGC​AGC​TTC​TC

### 2.6 Chronic photoaging model and experimental grouping

HDFs were seeded in 6-well plates (Corning Incorporated, Corning, NY, United States), pre-incubated with 50 μg/mL 3D-Exo or 3D-circ-Exo for 24 h, covered with a thin layer of PBS, and irradiated with UVA (365 nm peak, 5 J/cm^2^/d, 2.78 mW/cm^2^) for 7 days. Groups included: light-shielded control (NC), UVA-irradiated control (Control), 3D-Exo + UV, and 3D-circ-Exo + UV.

### 2.7 β-Galactosidase staining

Senescence-associated β-galactosidase activity was assessed using a staining kit (Beyotime Biotechnology, Shanghai, China) according to the manufacturer’s protocol. Stained cells were incubated overnight at 37°C (CO_2_-free) and imaged using an IX73 inverted microscope (Olympus Corporation, Tokyo, Japan).

### 2.8 Western blotting

Cells and exosomes were lysed in RIPA buffer (Beyotime Biotechnology, Shanghai, China) containing protease inhibitors (Roche Diagnostics, Basel, Switzerland). Protein concentration was determined by BCA assay (Beyotime Biotechnology, Shanghai, China). Equal protein amounts were separated on 10% SDS-PAGE gels, transferred to PVDF membranes, and probed overnight at 4°C with primary antibodies: Anti-P53 (Proteintech, Rosemont, IL, United States, #60283-2-Ig), Anti-P21 (Proteintech, Rosemont, IL, United States, #10355-1-AP), Anti-P16 (Proteintech, Rosemont, IL, United States, #10883-1-AP), Anti-Collagen I (Proteintech, Rosemont, IL, United States, #14695-1-AP), Anti-Elastin (Abcam, Cambridge, UK, #ab307150), Anti-β-actin (Proteintech, Rosemont, IL, United States, #66009-1-Ig). Membranes were incubated with HRP-conjugated secondary antibodies at room temperature for 1 h. Bands were visualized using ECL reagent (Proteintech, Rosemont, IL, United States) and quantified with ImageJ.

### 2.9 RNA extraction and qPCR

Total RNA was extracted using the EZ-press RNA Purification Kit (EZBioscience, Roseville, MN, United States). Exosomal RNA was isolated using the Exosomal RNA Purification Kit (EZBioscience, Roseville, MN, United States). cDNA was synthesized using the EasyScript All-in-One cDNA Synthesis Kit (TransGen Biotech, Beijing, China), and qPCR was performed using PerfectStart Green qPCR Mix (TransGen Biotech, Beijing, China) on a LightCycler^®^ 96 System (Roche Diagnostics, Basel, Switzerland). RNA concentration was measured using a NanoDrop One Microvolume UV-Vis Spectrophotometer (Thermo Fisher Scientific, Waltham, MA, United States). Relative expression was calculated using the 2^−ΔΔCT^ method (primers in Table 1).

### 2.10 Statistical analysis

Data are presented as mean ± SD from 3 independent biological replicates. Variance homogeneity was assessed using Levene’s test (α = 0.05); datasets failing this test were log-transformed before re-evaluation. For t-tests, we specified independent samples with Welch’s correction for unequal variances. For ANOVA, *post hoc* analyses used Fisher’s LSD (homogeneous variances) or Dunnett’s T3 (heterogeneous variances) with Bonferroni-adjusted p-values. Effect sizes (η^2^ for ANOVA; Cohen’s d for t-tests) are reported alongside exact p-values. Statistical analyses used SPSS 20.0. Significance thresholds: *p ≤ 0.05, **p ≤ 0.01, ***p ≤ 0.001, ****p ≤ 0.0001; NS = not significant.

## 3 Results

### 3.1 Construction and validation of 3d-circ-exo

#### 3.1.1 Phenotypic characterization and multilineage differentiation of hADSC

**FIGURE 1:**
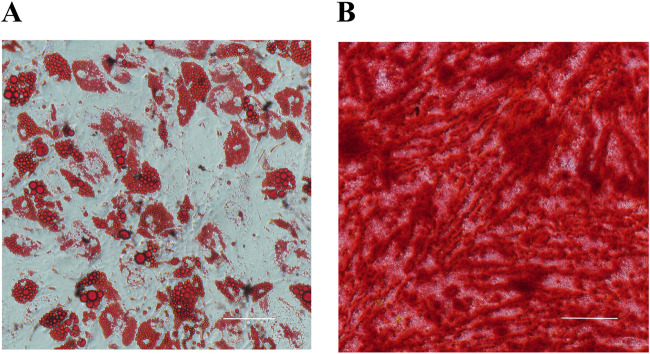
Multilineage differentiation of hADSCs (n = 3). **(A)** Adipogenic differentiation (Oil Red O staining). **(B)** Osteogenic differentiation (Alizarin Red staining).

Primary hADSCs isolated from donor adipose tissue displayed a spindle-shaped morphology. Flow cytometry confirmed their phenotype, showing positive expression for CD90 and CD105, and negative expression for CD34 and CD45 [as previously validated in Zhang et al. (2022)]. Following induction, hADSCs differentiated into adipocytes (verified by Oil Red O staining of lipid droplets) and osteocytes (verified by Alizarin Red staining of mineralized nodules) (Figures 1A, B), confirming their multipotency.

#### 3.1.2 Construction and validation of circ_0011129 overexpression vector

**FIGURE 2:**
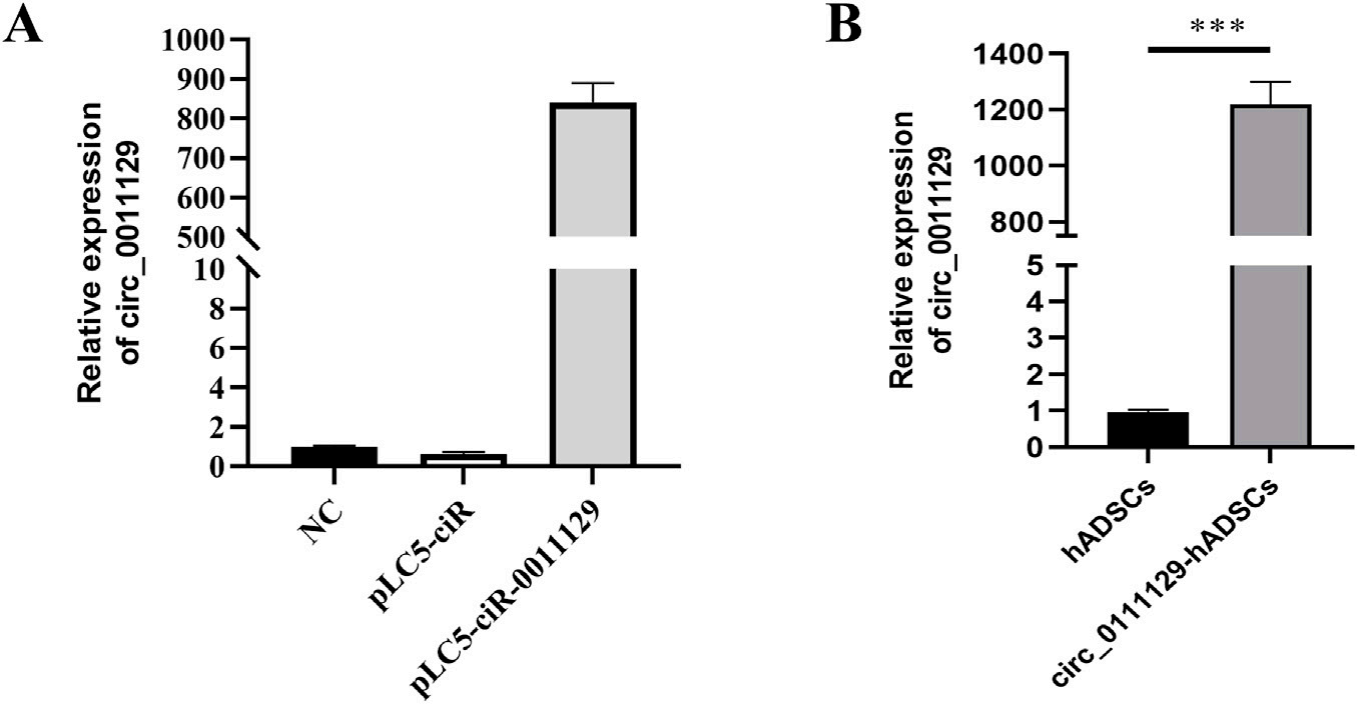
Construction and validation of circ_0011129 overexpression vector. **(A)** circ_0011129 expression in empty vector and plasmid vector groups. **(B)** Relative circ_0011129 expression in 3D-hADSCs and 3D-circ_0011129-hADSCs (n = 3). NS: not significant; ***p ≤ 0.001.

qPCR analysis showed significantly higher circ_0011129 expression in HEK-293T cells transfected with the pLC5-circ_0011129 plasmid compared to negative control (NC) and empty vector (pLC5-ciR) groups (Figure 2A). Lentiviral transduction of 3D-hADSCs also resulted in significant circ_0011129 upregulation compared to untransfected cells (p < 0.001, Figure 2B), confirming successful generation of circ_0011129-overexpressing 3D-hADSCs.

#### 3.1.3 Circular RNA validation and stability analysis of 3D-circ-Exo

**FIGURE 3:**
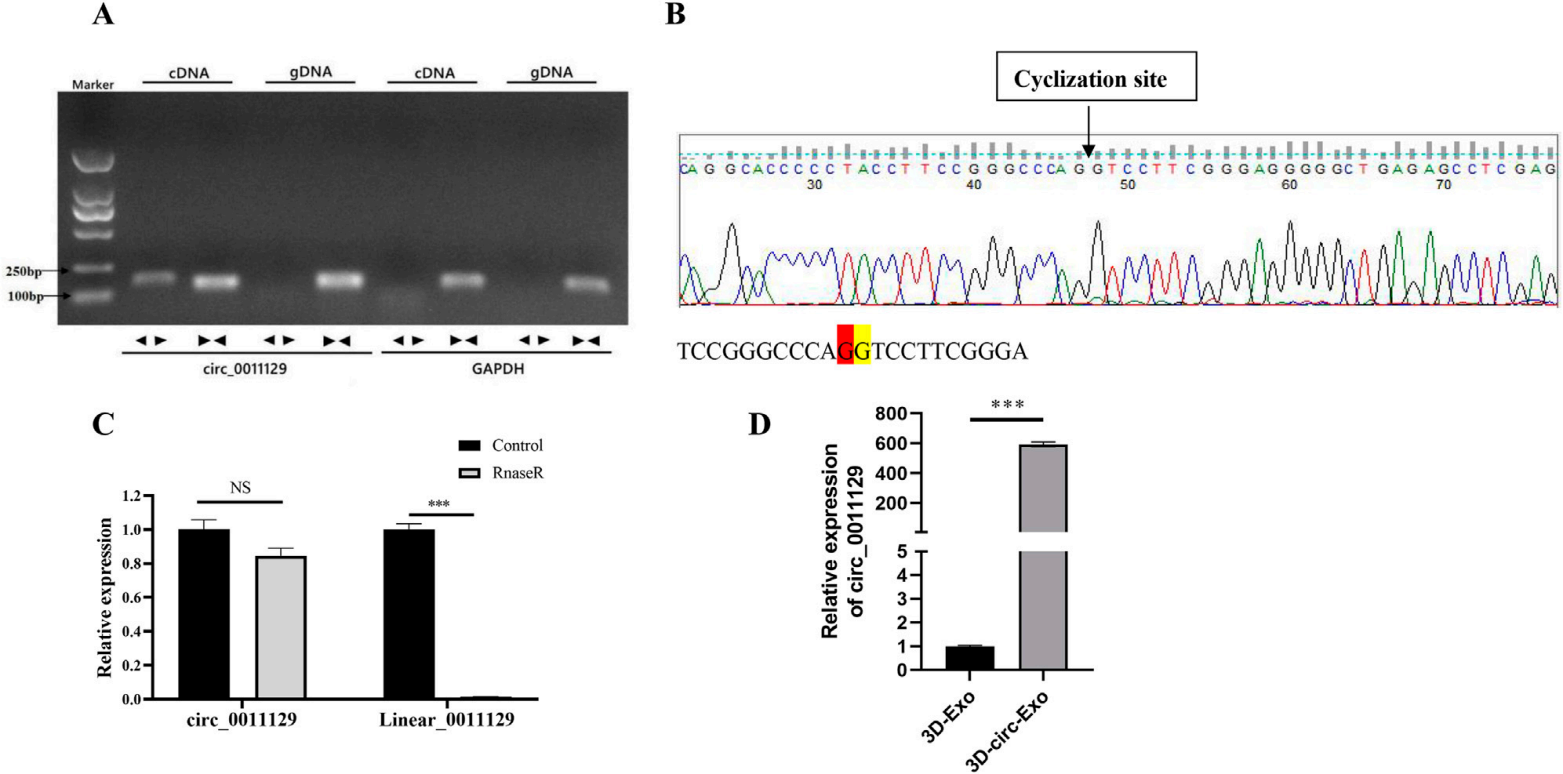
Circular RNA validation and stability analysis of 3D-circ-Exo (n = 3). **(A)** Amplification using divergent/convergent primers. **(B)** Sanger sequencing of back-splice junction. **(C)** RNase R stability assay. **(D)** qRT-PCR of circ_0011129 levels. NS: not significant; ***p ≤ 0.001.

Divergent primers amplified circ_0011129 only from cDNA, while convergent primers amplified it from both cDNA and gDNA, confirming its circular structure (Figure 3A). Sanger sequencing matched circ_0011129s back-splice junction to circBase records (Figure 3B). RNase R digestion demonstrated the resistance of circ_0011129 in 3D-circ-Exo to degradation, contrasting with its linear counterpart (Figure 3C). qPCR confirmed significantly higher circ_0011129 levels in 3D-circ-Exo compared to control exosomes (3D-Exo) (p < 0.001, Figure 3D). These results validated the successful loading and structural integrity of circ_0011129 in 3D-circ-Exo.

### 3.2 Establishment and validation of chronic photoaging dermal fibroblast model

#### 3.2.1 Senescence phenotype validation

**FIGURE 4:**
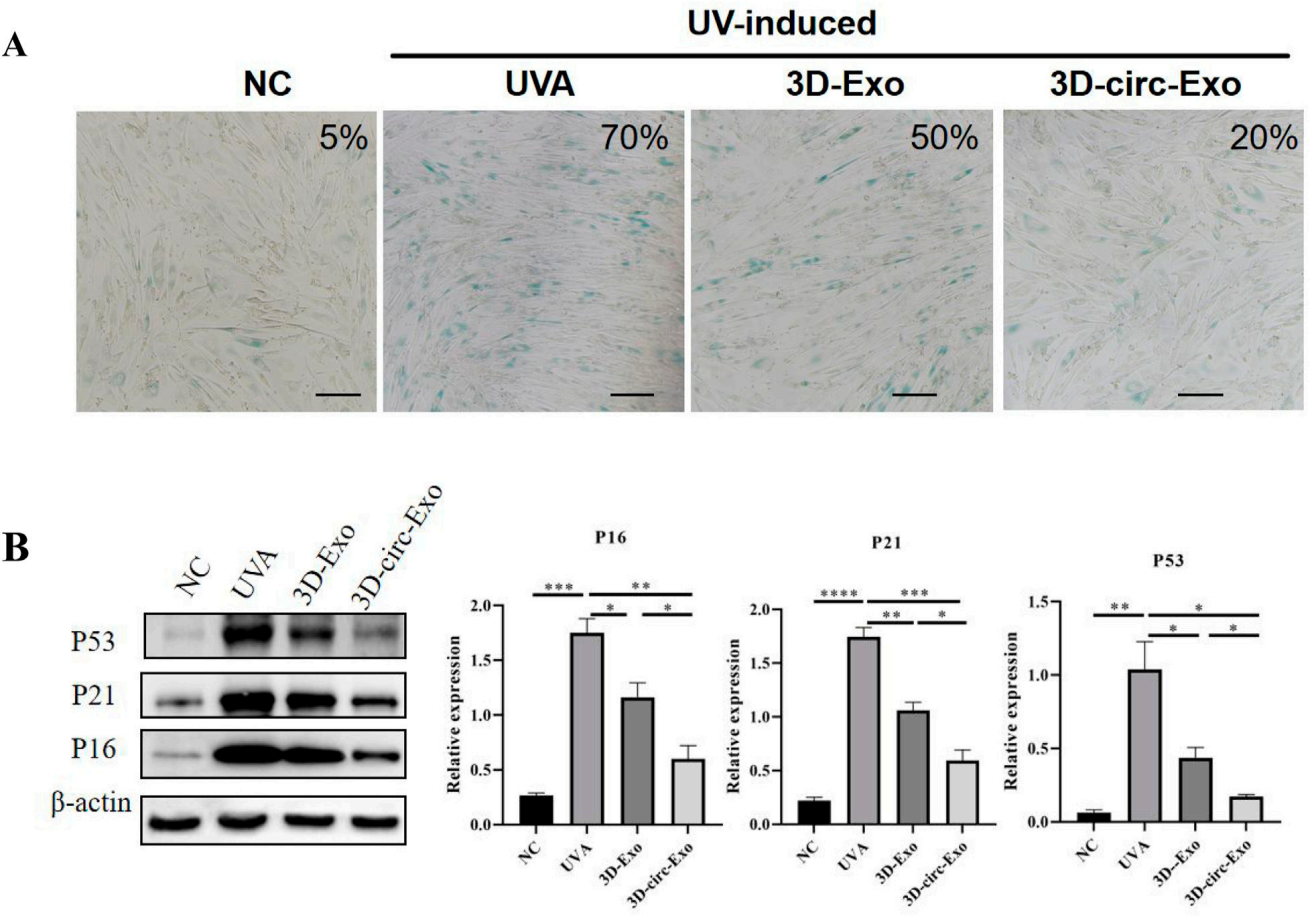
Establishment and validation of chronic photoaging HDFs model (n = 3). **(A)** SA-β-gal staining. **(B)** Western blot of p53, p21, p16 expression across groups. NS: not significant; *p ≤ 0.05; **p ≤ 0.01; ***p ≤ 0.001; ****p ≤ 0.0001.

After 7 days of UVA irradiation (5 J/cm^2^/day), senescence-associated β-galactosidase (SA-β-gal) staining showed a significant increase in positive cells compared to the non-irradiated (NC) group (Figure 4A), indicating induced senescence.

#### 3.2.2 Cell cycle regulatory protein Detection

Western blot analysis revealed significant upregulation of p53, p21, and p16 protein levels in UVA-irradiated HDFs compared to NC (p < 0.01, Figure 4B), confirming cell cycle arrest and DNA damage response. These findings validate the chronic photoaging model.

### 3.3 Anti-photoaging effects of 3d-circ-exo

#### 3.3.1 3D-circ-Exo inhibits cellular senescence

SA-β-gal staining showed that both 3D-Exo and 3D-circ-Exo significantly reduced the proportion of senescent cells compared to the UVA group, with 3D-circ-Exo exhibiting greater inhibition (Figure 4A). Consistent with this, 3D-circ-Exo significantly downregulated p53, p21, and p16 protein expression compared to both UVA and 3D-Exo groups (p < 0.05, Figure 4B).

#### 3.3.2 3D-circ-Exo protects extracellular matrix proteins

**FIGURE 5:**
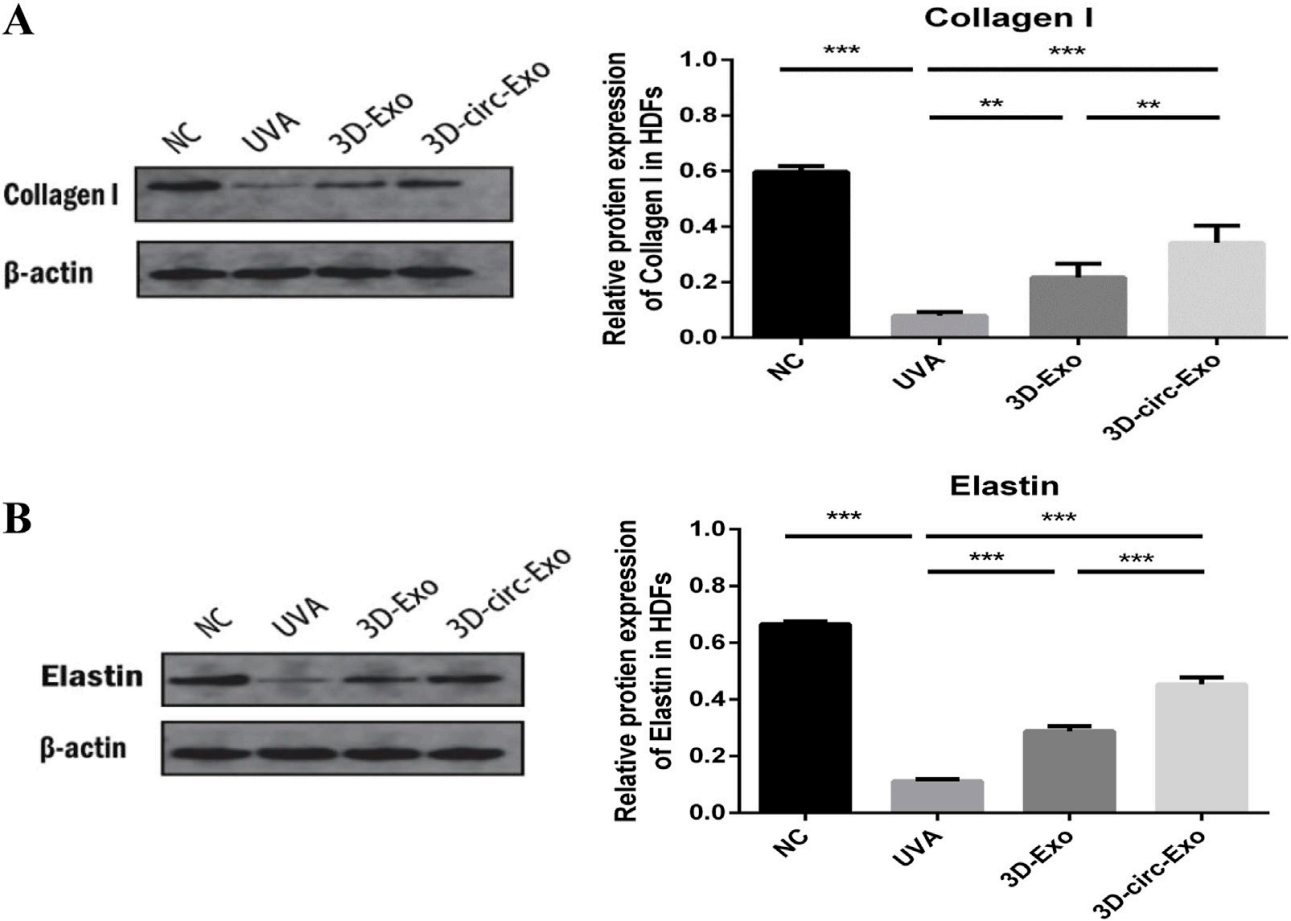
3D-circ-Exo protects extracellular matrix proteins in photoaged HDFs (n = 3). **(A)** Western blot analysis of collagen I expression in chronic photoaging HDFs. **(B)** Western blot analysis of elastin expression. NS: not significant; *p ≤ 0.05; **p ≤ 0.01; ***p ≤ 0.001.

Western blot analysis showed that both 3D-Exo and 3D-circ-Exo significantly upregulated collagen I and elastin expression compared to the UVA group (p < 0.01, Figure 5A). Notably, 3D-circ-Exo induced a significantly stronger upregulation of these matrix proteins than 3D-Exo (p < 0.01, Figure 5B).

## 4 Discussion

Photoaging, the primary cause of extrinsic skin aging, significantly impacts physical appearance, psychological wellbeing, and increases the risk of sunlight-related skin diseases, including malignancies (Kimura et al., 2025; Wenande et al., 2025; Zundell et al., 2025). Early intervention is therefore clinically important. The pathological hallmarks of photoaging—collagen degradation and abnormal elastin accumulation—stem from UV-induced oxidative stress, inflammation, and protease dysregulation. In this study, we integrated 3D culture with exosome delivery to develop functionalized exosomes loaded with circ_0011129 (3D-circ-Exo). We demonstrated that 3D-circ-Exo synergistically modulates cell cycle arrest and extracellular matrix metabolism, effectively reducing senescence and matrix degradation in a chronic photoaging fibroblast model. This approach offers a novel multi-target strategy for photoaging intervention.

CircRNAs show therapeutic potential due to their stability and nuclease resistance. However, poor *in vivo* delivery efficiency limits their clinical translation (Shi et al., 2025; Alshehry et al., 2025). Exosomes, with their low immunogenicity, targeting capacity, and stability, represent ideal RNA carriers (Zhang et al., 2025; Cai et al., 2025). We innovatively combined 3D culture with exosome delivery, using hADSC-derived exosomes as carriers for circ_0011129 to construct 3D-circ-Exo. Divergent primer amplification, sequencing, and RNase R digestion confirmed the circular structure of circ_0011129 within 3D-circ-Exo, its superior stability over linear RNA (p < 0.001), and resistance to degradation. 3D culture not only increases exosome yield but also enhances their bioactivity, as seen in other models (Pourhadi et al., 2024). Comparing 3D-Exo and 3D-circ-Exo revealed a synergistic effect: 3D-circ-Exo further reduced SA-β-gal-positive cells compared to 3D-Exo alone. This suggests that bioactive exosome components (e.g., anti-inflammatory factors) and circ_0011129s regulatory function complementarily counteract photoaging, establishing a scalable platform for circRNA-exosome therapeutics.

**FIGURE 6:**
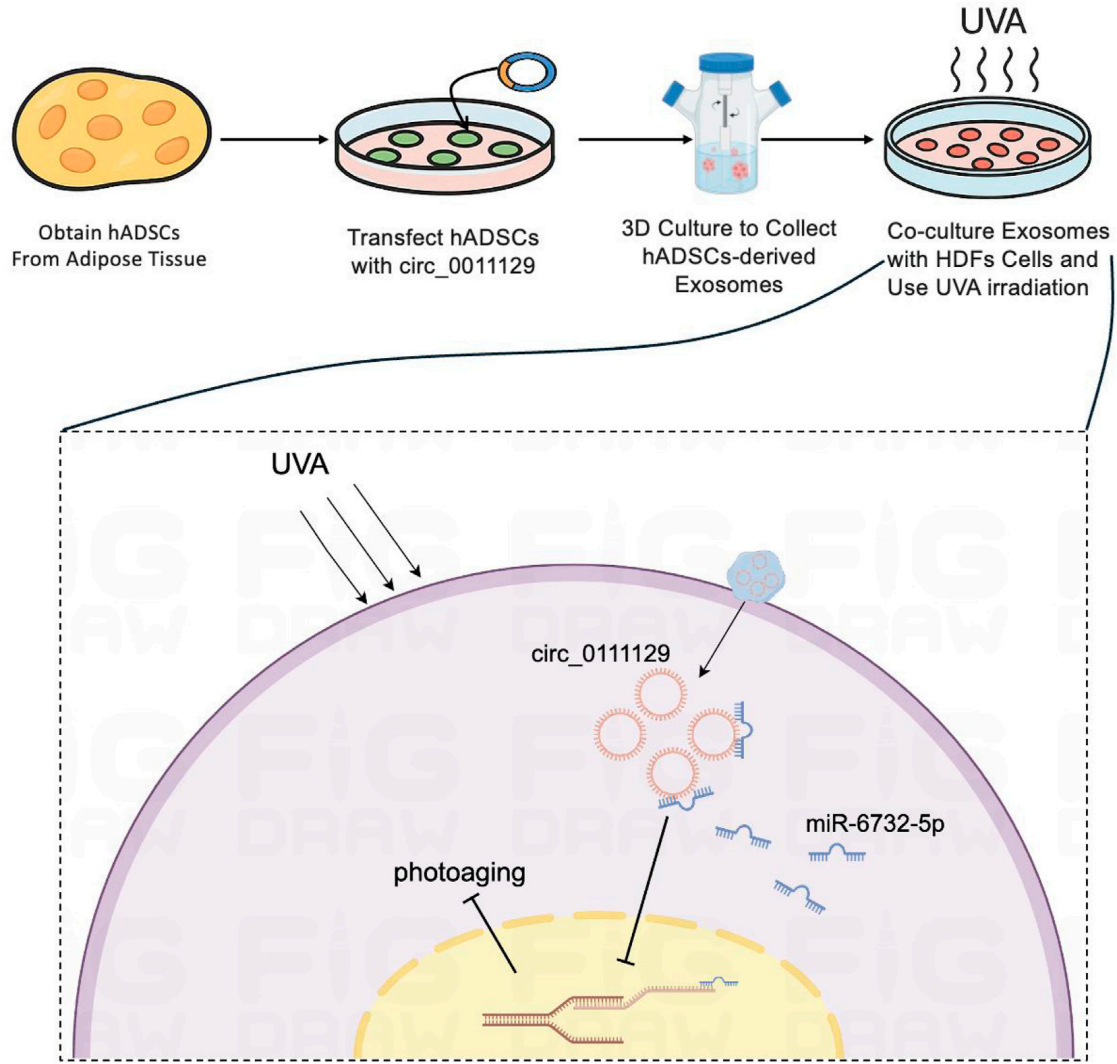
Diagrammatic representation of the inhibitory mechanism targeting cellular photoaging.

In our chronic photoaging model, UVA irradiation activated the p53/p21/p16 pathway, inducing HDF cell cycle arrest (Figure 4B), while downregulating collagen I and elastin expression (Figures 5A,B). This highlights the link between cellular senescence and matrix dysregulation in photoaging. Current interventions often target single pathways (e.g., MMP inhibitors) and fail to address both senescence and matrix degradation simultaneously. Notably, the anti-senescence effect of 3D-Exo alone aligns with established evidence that MSC-derived exosomes intrinsically deliver protective cargo (e.g., antioxidants, growth factors) (Wang et al., 2024; Liu et al., 2024), further amplified by 3D-culture-enhanced bioactivity (Lee and Lee, 2024). Crucially, our study demonstrates that 3D-circ-Exo exerts dual regulatory effects on these processes. Specifically, 3D-circ-Exo further reduced SA-β-gal-positive cells and significantly downregulated p53, p21, and p16 expression (p < 0.01) compared to 3D-Exo. It also more potently upregulated collagen I and elastin levels (p < 0.01). These results indicate that 3D-cultured exosomes inherently possess anti-photoaging activity, which is enhanced by circ_0011129 loading, suggesting synergistic regulation. This synergy may arise from: (1) Anti-inflammatory factors and growth factors in 3D-hADSC-exosomes inhibiting UV-induced oxidative stress and MMP activation (Park et al., 2023; Yan et al., 2023; Zou et al., 2024); and (2) circ_0011129 directly regulating collagen synthesis and elastin stability by sponging miR-6732-5p (Lin et al., 2020; Zhang et al., 2022), complementing the protective effects of exosomes (Figure 6).

Despite the promising anti-photoaging effects of 3D-circ-Exo, clinical translation faces challenges. First, large-scale exosome production and standardized quality control require optimization. While 3D culture improves yield, industrial-scale applications encounter cost and stability hurdles (Codrich et al., 2021; Nooshabadi et al., 2020; Thongsit et al., 2025; Jang et al., 2024). Second, the epidermal barrier may limit exosome penetration, necessitating transdermal delivery technologies (e.g., microneedles, nanocarriers) for enhanced targeting. Finally, long-term safety assessments, particularly regarding potential immunogenicity and off-target effects of circRNA-loaded exosomes, need validation in large animal models.

In conclusion, we successfully constructed functionalized circ_0011129 carriers (3D-circ-Exo) by integrating 3D culture with exosome delivery. 3D-circ-Exo exhibits superior anti-photoaging efficacy compared to 3D-Exo alone, demonstrating the synergistic potential of combining exosomes with circRNA. This strategy provides a multi-target approach for skin photoaging and lays a foundation for clinical translation of circRNA-exosome therapies. Future research will focus on *in vivo* validation and delivery system optimization to advance this strategy towards clinical application.

## Data Availability

The datasets presented in this study can be found in online repositories. The names of the repository/repositories and accession number(s) can be found below: Zenodo repository under the accession number DOI 10.5281/zenodo.16931506.
